# The Effect of Cross-linking Efficiency of Drug-Loaded Novel Freeze Gelated Chitosan Templates for Periodontal Tissue Regeneration

**DOI:** 10.1208/s12249-020-01708-x

**Published:** 2020-06-16

**Authors:** Syed Saad B. Qasim, Liebert Parreiras Nogueria, Amr S. Fawzy, Umer Daood

**Affiliations:** 1 Department of Biomaterials, Institute of Clinical Dentistry, University of Oslo, Oslo, Norway; 2 Department of Bioclinical Sciences, Faculty of Dentistry, Kuwait University, PO-Box 24923, 11310 Safat, Kuwait; 3 UWA Dental School, University of Western Australia, 17 Monash Avenue, Nedlands, WA 6009 Australia; 4 Clinical Dentistry Division, Restorative Division, School of Dentistry, International Medical University Kuala Lumpur, 126, Jalan Jalil Perkasa 19, Bukit Jalil 57000, Wilayah Persekutuan, Kuala Lumpur, Malaysia

**Keywords:** chitosan, doxycycline hyclate, glutaraldehyde, functionally graded, periodontitis

## Abstract

Innovative strategies for periodontal regeneration have been the focus of research clusters across the globe for decades. In order to overcome the drawbacks of currently available options, investigators have suggested a novel concept of functionally graded membrane (FGM) templates with different structural and morphological gradients. Chitosan (CH) has been used in the past for similar purpose. However, the composite formulation of composite and tetracycline when cross-linked with glutaraldehyde have received little attention. Therefore, the purpose of the study was to investigate the drug loading and release characteristics of novel freeze gelated chitosan templates at different percentages of glutaraldehyde. These were cross-linked with 0.1 and 1% glutaraldehyde and loaded with doxycycline hyclate. The electron micrographs depicted porous morphology of neat templates. After cross-linking, these templates showed compressed ultrastructures. Computerized tomography analysis showed that the templates had 88 to 92% porosity with average pore diameter decreased from 78 to 44.9 μm with increasing concentration. Fourier transform infrared spectroscopy showed alterations in the glycosidic segment of chitosan fingerprint region which after drug loading showed a dominant doxycycline spectral composite profile. Interestingly, swelling profile was not affected by cross-linking either at 0.1 and 1% glutaraldehyde and template showed a swelling ratio of 80%, which gained equilibrium after 15 min. The drug release pattern also showed a 40 μg/mL of release after 24 h. These doxycycline-loaded templates show their tendency to be used in a functionally graded membrane facing the defect site.

## INTRODUCTION

Innovative strategies for periodontal regeneration have been the focus of research clusters across the globe for decades (1–3). A number of different treatment modalities are available to clinicians for regenerating lost tooth supporting structures. Amongst the available options, a guided tissue regenerative (GTR) membrane is commonly used. These membranes are either used alone or in combination with other bone substitutes (4). Therefore, they are able to heal defects created by chronic inflammatory lesions like periodontitis. However, the evidence to support predictable regeneration whilst using these templates still require further development (5). Some biomechanical, functional and structural limitations with respect to handling and degradation are still unmet (6). Therefore, a concept of functionally graded membrane (FGM) with different structural and morphological gradients has been proposed by investigators (6–10).

**Fig. 1:**
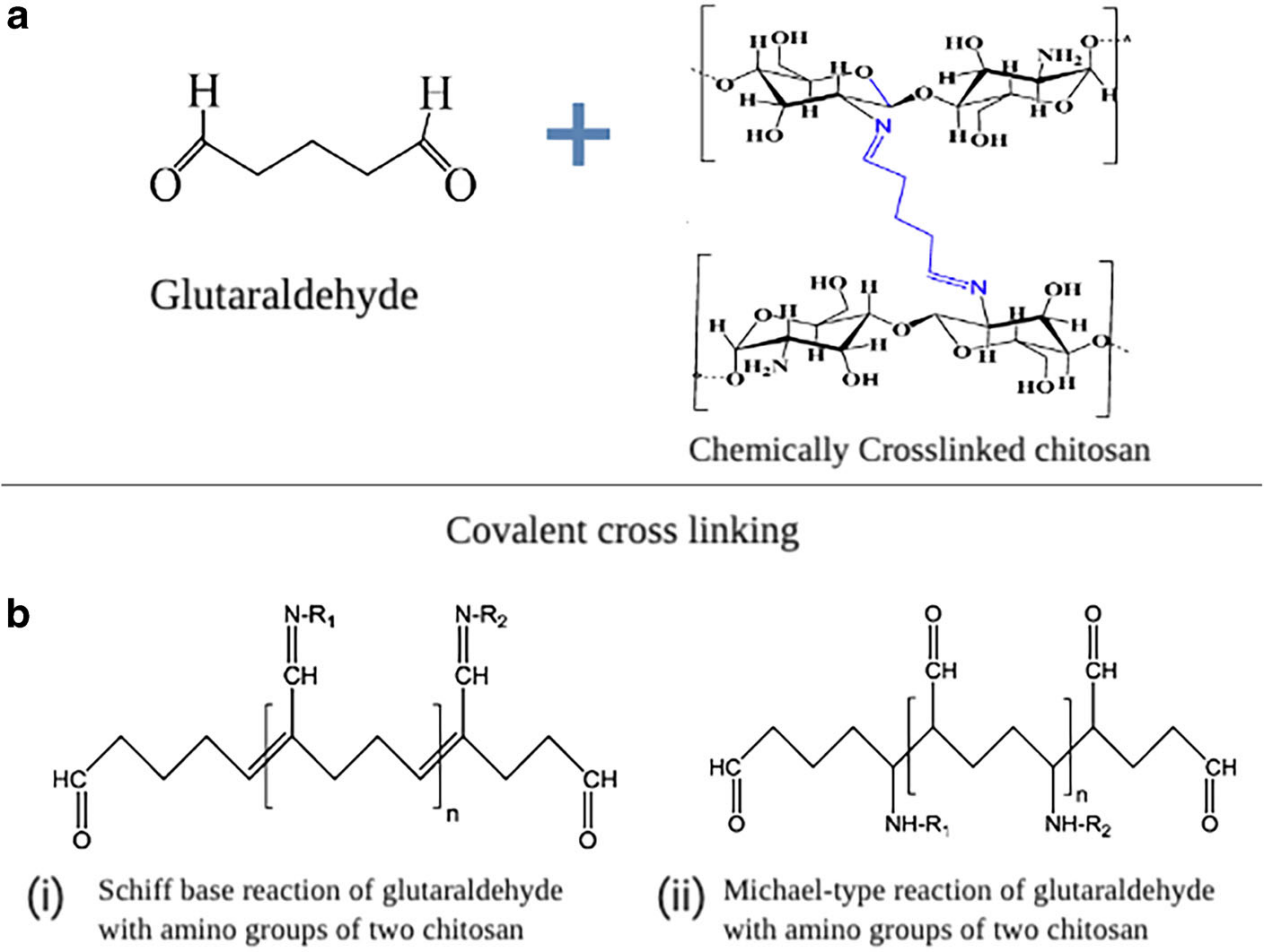
Diagrammatic illustration of possible interactions of chitosan and glutaraldehyde. **a** Chitosan cross-linked with glutaraldehyde chemically. **b**(i) and (ii) Covalent linkages of chitosan and glutaraldehyde *via* Schiff’s base and Michael type reaction

In order to develop these FGM, different natural and synthetic polymers have been proposed in combination with different bioactive molecules. Hydrogels have been investigated as scaffolds and are suggested to play a crucial role due to the diverse nature of these systems. Moreover, in order to tailor the physio-chemical and mechanical characteristics, the convenience of cross-linking for controlling degradation and loading biomolecules like drugs can be incorporated within hydrogels as well (6). One of the most researched biopolymer for tissue engineering applications is chitosan (CH) (Fig. 1). It is derived from chitin, which is the second most abundantly available amino polysaccharide in nature. CH has been used to fabricate porous membranes for tissue regeneration alone and in combination with either calcium phosphates or antimicrobial drugs (11,12). Porosities have been incorporated by freeze casting CH solutions. By harnessing the freezing rate of the ice crystals, porous morphology has been achieved (10).

Localized delivery of drugs has an effect on its efficacy, using lower dosage values, and a more controlled release profile can be achieved (13). Tetracycline has been used in different therapeutic forms due to their ability to reduce microbial burden, inhibiting collagenase activity and bone loss (14). Composite formulations of CH and tetracycline have also been reported in the past (15,16). Due to the polycationic nature of CH, drug loading on hydrogels is assisted by using cross-linking agents such as genipin or glutaraldehyde. Amongst the available cross-linkers, glutaraldehyde has been heavily researched (Fig. 1) (17,18). The degree of cross-linking of CH is dependent on the degree of deacetylation only (19). Although there have been speculations about glutaraldehyde being relatively toxic, there are studies which support that CH can be conveniently cross-linked with glutaraldehyde (20,21). However, reports about the effect of cross-linking efficiency on drug entrapment percentage and release profile of freeze casted porous scaffolds are still elusive. Therefore, the purpose of the study was to investigate the drug loading and release characteristics of freeze casted CH scaffolds at different percentages of glutaraldehyde. Our hypothesis is that freeze casted CH membranes can be conveniently loaded with doxycycline hyclate and form an integral element of a functionally graded membrane for periodontal regeneration.

## MATERIALS AND METHODS

The experimental procedures were performed in two parts. First step was to prepare freeze casted CH scaffolds at different percentages and cross-link with glutaraldehyde at 0.1 and 1%. The second phase was spent on drug loading and studying the release profile. Chitosan (CH) (ChitoClear® Iceland) (molecular weight 133,760 Da, degree of de-acetylation = 96.6%), doxycycline hyclate (Sigma-Aldrich), acetic acid, glutaraldehyde (Sigma-Aldrich), sodium hydroxide (NaOH) (VWR, Chemical), ethanol, phosphate-buffered tablets (Tablets, Sigma-Aldrich), glycerol (Fisher Scientific, UK).

### Fabrication of Templates

Freeze casting has been reported by Qasim *et al.*, previously (10). Briefly 2, 4 and 6% solutions of CH were made. Initially, CH was dissolved in distilled water (29.64 mL) for 30 min, and then, acetic acid (360 μL) was added dropwise to achieve a 0.2 M (M) concentration. The solutions were stirred at 37°C for 2 h. These were then poured onto plastic Petri dishes to be stored at 4°C for 12 h. The plastic Petri dishes were then transferred to − 20°C freezer and left for another 12 h to freeze. A neutralizing solution of 3 M sodium hydroxide (NaOH) and ethanol in a ratio of 1:1 were pre-freezed and the frozen discs were submerged in this solution and left at − 20°C. After 12 h, these were taken out and dried out using 70, 80, 90, 95 and 100% ethanol before immersing them in a 1:10 solution of glycerol and distilled water. The templates were dried at 40°C for 30 min before storing them in sealed bags.

### Cross-linking and Drug Loading

Chitosan solutions at concentrations of 2, 4 and 6% (30 mL) were prepared in the manner described above. Once the acetic acid was added dropwise, two different concentrations of glutaraldehyde cross-linking solutions were prepared at 0.1 and 1% (1 mL). These cross-linking agents were added dropwise in the chitosan solutions and the templates were freeze gelled following the protocol mentioned previously. Once the templates were dried, they were carefully rinsed with distilled water at room temperature (24°C ± 2). After each wash, fresh water was used to rinse the template again for 24 h. Once cross-linking protocol was completed, the templates were dried at 40°C for 30 min. Figure 1 shows the possible chemical reactions of chitosan and glutaraldehyde. The neat and cross-linked templates were loaded with 125 mg/mL of doxycycline hyclate dissolved in methanol (Sigma-Aldrich, UK). All specimens were immersed in 1 mL of the drug solution for 24 h and then dried in an oven at 45°C and placed in a desiccator before testing.

### Scanning Electron Microscopy Analysis

Scanning electron microscopy (SEM) (Hitachi Analytical Table Top Microscope / Benchtop SEM TM3030) at a voltage of 15 kV was performed after the samples were mounted on cylindrical aluminium stubs covered with double-sided carbon adhesive dots and were sputter coated under vacuum with gold (Cressington 108A Auto Sputter coaters) to investigate the porous morphology in cross section.

### Nano-computerized Tomography

The pore size, porosity percentage and structure thickness were calculated using nano-computerized tomography (nCT) (SKYSCAN 2211 Bruker***,*** Kontich, Belgium). The images were acquired with a final isotropic voxel size of 750 nm, at camera binning of 1 × 1, 34 kV accelerating voltage, 340 μA current and with no physical filter placed in front of the beam outlet. Samples were programmed to be rotated around 360° about the vertical axis at a step size of 0.31°, with exposure time of 1000 ms per projection taking an average of two frames and making a total of 1 h acquisition time. Image reconstruction was conducted using NRecon software (Bruker, Belgium).

### Fourier Transform Infrared Spectroscopy

Fourier transform infrared (FTIR) spectroscopy (PerkinElmer, Waltham, MA, USA) was done for the neat, cross-linked and drug-loaded samples using attenuated total reflectance (ATR) accessory equipped with a diamond ATR crystal. Spectral profiles were conducted in the mid-infrared region (600 to 3500 cm^−1^) at a resolution of 4 cm^−1^ by accumulating 32 scans. Obtained spectra were processed using Spectrum™ 10 software.

### Swelling Profile and Drug Entrapment Efficiency

Specimens (6 mm in diameter) were dried and weighed. These were then stored in PBS inside a temperature-controlled incubator at 37°C. At pre-set time points, specimens were retrieved from the PBS and any excess water was removed using tissue paper. Time points used were 0, 15 min, 30 min, 1 h, 2 h and 24 h. The swelling ratio was calculated using the formula (9).

$$\mathrm{Swelling}\ \mathrm{ratio}\%(Q)=\left({W}_{\mathrm{w}}-{W}_{\mathrm{d}}\ \right)/{W}_{\mathrm{d}}\times 100$$

Where dry weight was noted as *W*_d_ and wet weight was noted as *W*_w._

Drug entrapment efficiency was conducted by weighing the templates before and after drug loading in dry condition by using the formula (22).

$$\mathrm{Amount}\ \mathrm{of}\ \mathrm{drug}={W}_{\mathrm{d}}/{W}_{\mathrm{t}}\times 100$$

where *W*_d_ was the weight of drug in the membranes and *W*_t_ was the theoretical weight of the drug loaded in the membrane.

### *In Vitro* Drug Release Profile Conventional Dialysis Sac Method

The *in vitro* drug release profile was performed by placing the specimens inside a dialysis membrane (MEMBRA-CEL® USA) (14,000 Da) containing 5 mL of PBS. The two ends of the dialysis sac were tightly sealed with clamps. The sac was then placed inside a 1000-mL beaker containing 500 mL of PBS (pH = 7.4) at 37°C under constant magnetic stirring (300 rpm) and placed inside an oven (37°C) during the 24 h of analysis. All experiments were conducted in triplicate. At pre-set time points of 15, 30, 60, 120 min and 24 h, 1-mL of medium was retrieved. The experimental conditions were maintained at an equilibrium state by replacing this with fresh pre-warmed PBS. The drug concentration was determined by using UV-Vis Spectroscopy (PerkinElmer Lambda 25 UV/° Vis Systems, USA). A calibration curve was made using 5 parameter logistics curve fit as reported by Findlay and Dillard (23). Drug concentration was measured at 268 nm. All data were analysed using Graphpad Prism software (version 8.0). Cumulative release percentage was using the calculated formula reported previously (24)**.**

### Statistical Analysis

Unless otherwise stated, all experiments were conducted in triplicate. The data shown refers to mean ± standard deviation (SD). Statistically significant differences were evaluated using a one-way ANOVA, followed by Tukey’s *post hoc* test. Results with *p* values of < 0.05 (α) were considered statistically significant. All data were analysed using Graphpad Prism 8.0 software.

## RESULTS

### Scanning Electron Microscopy

**Fig. 2:**
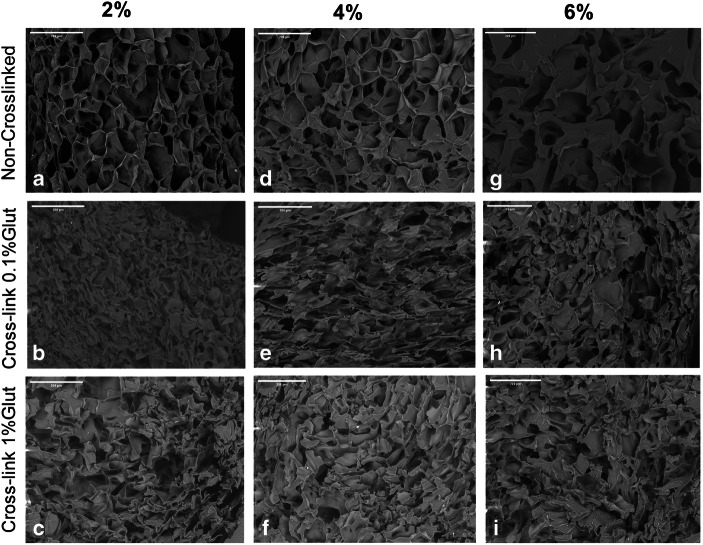
SEM images of freeze gelated 2, 4 and 6% chitosan cross-linked with 0.1 and 1% glutaraldehyde. All images are scaled at 200 μm

Cross-sectional SEM images of freeze gelated CH templates at 2, 4 and 6% are shown in Fig. 2, along with their cross-linked counterparts at 0.1 and 1%. Images show that the pores are uneven in structural morphology as compared to the cross-linked versions. Pores of non-cross-linked templates at 2% showed a more even distribution whereas at 4% and 6%, they seem spread out to some extent. They also depicted a heterogenous morphology made up of polyhedral pores. Similarly, the pore boundaries of 2% are sharper as compared to the other two concentrations. Cross-linking templates displayed pores that seem to be compressed. These flattened pores are evident in both concentrations of cross-linking agent at 0.1 and 1%. The neat templates show that some evidence of interconnectivity, which is more prominent in 2 and 4% CH templates.

### Multiscale Computerized Tomography

**Fig. 3:**
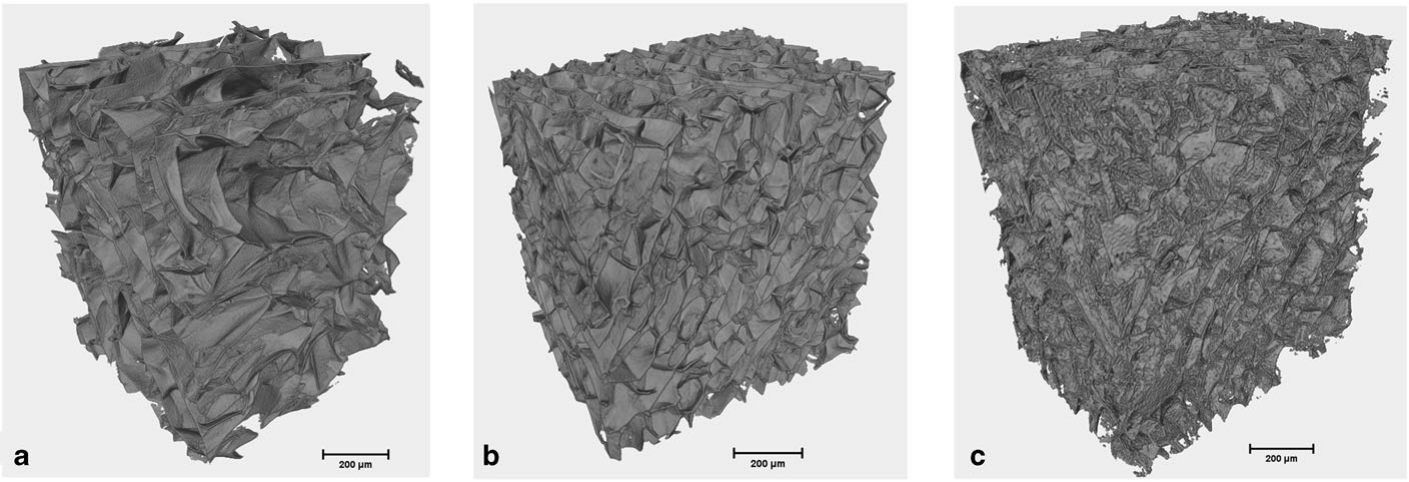
Computerized tomography images of templates prepared by 2, 4 and 6% chitosan. Images depicting the porous morphology achieved. All images are scaled at 200 μm

Data extracted from the computerized tomography (CT) of the templates show that highest porosity percentage was noted for 2% templates with porosity of 92.6% and an average pore diameter of 78 μm. Increasing the concentration to 6% resulted in reduction of the pore size to 45 μm and the porosity percentage was 90%. The morphometrical parameter in Fig. 3 shows three-dimensional sections of the template depicting the pore morphology.

### Fourier Transform Infrared Spectroscopy

**Fig. 4:**
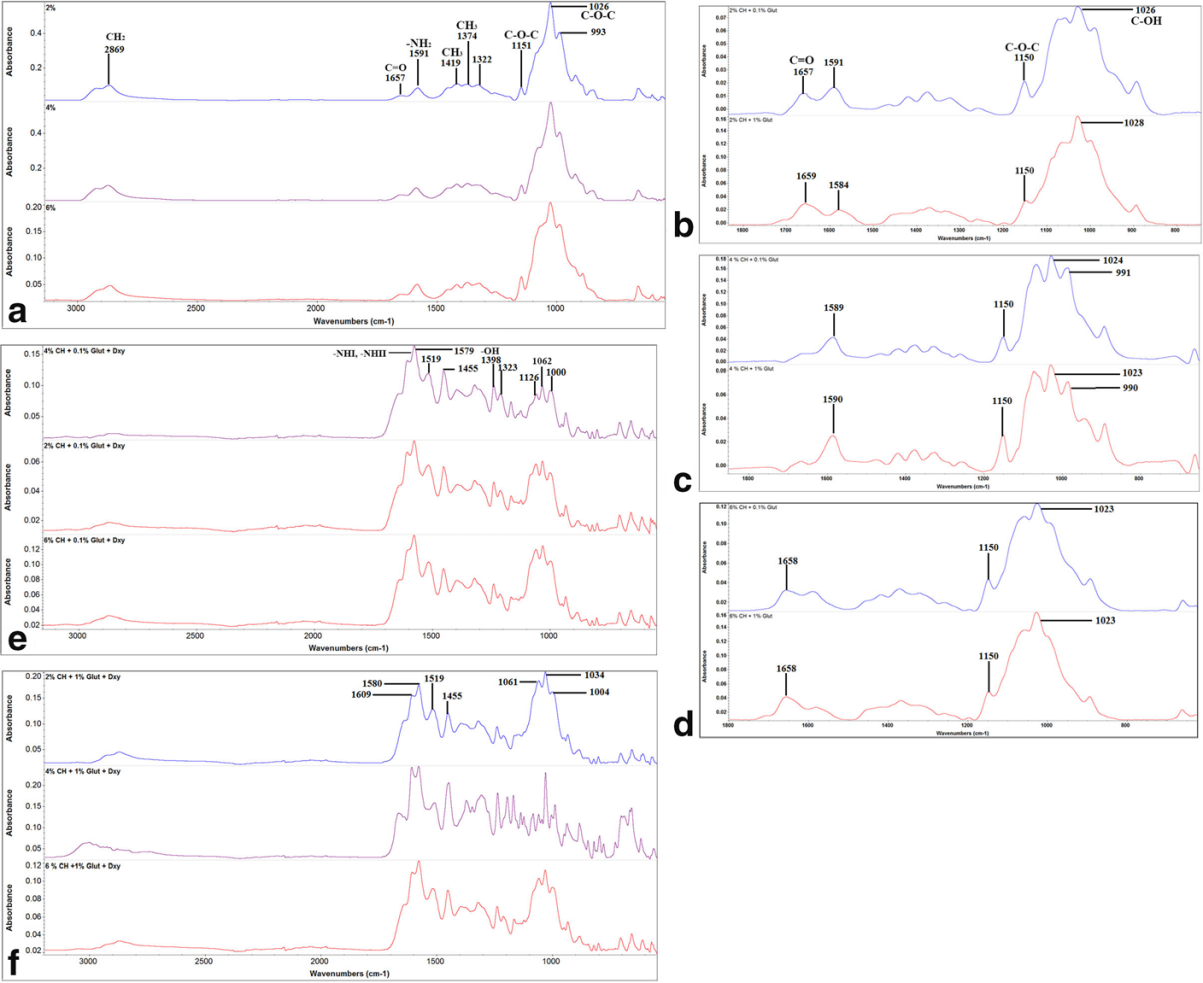
FTIR spectral profile of **a** neat chitosan scaffolds at 2, 4 and 6%. **b** The finger print region of 2% chitosan cross-linked at 0.1 and 1% glutaraldehyde; **c** 4% chitosan cross-linked at 0.1 and 1% glutaraldehyde; **d** 6% chitosan cross-linked at 0.1 and 1% glutaraldehyde; **e** 2, 4 and 6% chitosan cross-linked at 0.1% glutaraldehyde and loaded with doxycycline hyclate; **f** 2, 4 and 6% chitosan cross-linked at 1% glutaraldehyde and loaded with doxycycline hyclate

FTIR spectra of neat, cross-linked and drug-loaded samples are shown in Fig. 4a–d. The neat templates show typical spectral profile of CH molecular structure with C–H stretching vibrations occurring at 2869 cm^−1^. Amide I and II stretching and bending vibrations were noted at 1657 cm^−1^ assigned to C=O and 1591 cm^−1^ assigned to –NH_2_. The rocking and bending modes of C–H were noted at 1419 cm^−1^ and 1374 cm^−1^. The typical glycosidic linkages of pyranose *v*_3_ C–O–C and C–O stretching modes were also noted at 1151 cm^−1^ and 1026 cm^−1^ (Fig. 4a). Finger print region of the cross-linked templates are shown in Fig. 4b–d of 2, 4 and 6% CH. The change in the intensity and shifting of wavenumbers of amide I and II peaks to 1659, 1584 and 1589 cm^−1^, glycosidic segment to 1024, 1028 and 1023 cm^−1^. Drug-loaded template is shown in Fig. 4 e and f. Finger print regions of these spectra collected show the peaks pertaining to doxycycline hyclate at 1579, 1519, 1455, 1398, 1323, 1126, 1062 and 1000 cm^−1^.

### Swelling Profile

**Fig. 5:**
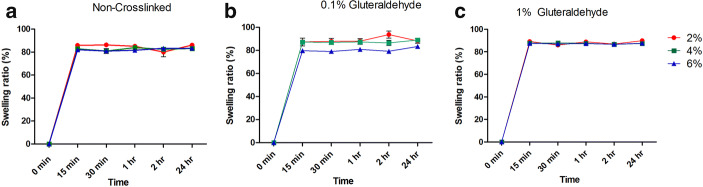
Swelling profile of 2, 4 and 6% chitosan and their cross-linked counterparts at 0.1 and 1% glutaraldehyde conducted at 0, 15, 30, 60, 120 min and 24 h. Samples tested in triplicate (*n* = 3) ± SEM

The swelling profile of non-cross-linked and cross-linked templates is shown in Fig. 5. All membranes achieve a state of equilibrium after 15 min of swelling. The neat membranes reached 80% swelling percentage regardless of the CH concentration. The cross-linked specimens at 0.1 and 1% also gained a swelling percentage of 80% and maintained it for the remaining time of the analysis.

### Percentage Drug Release and Entrapment Efficiency

**Fig. 6:**
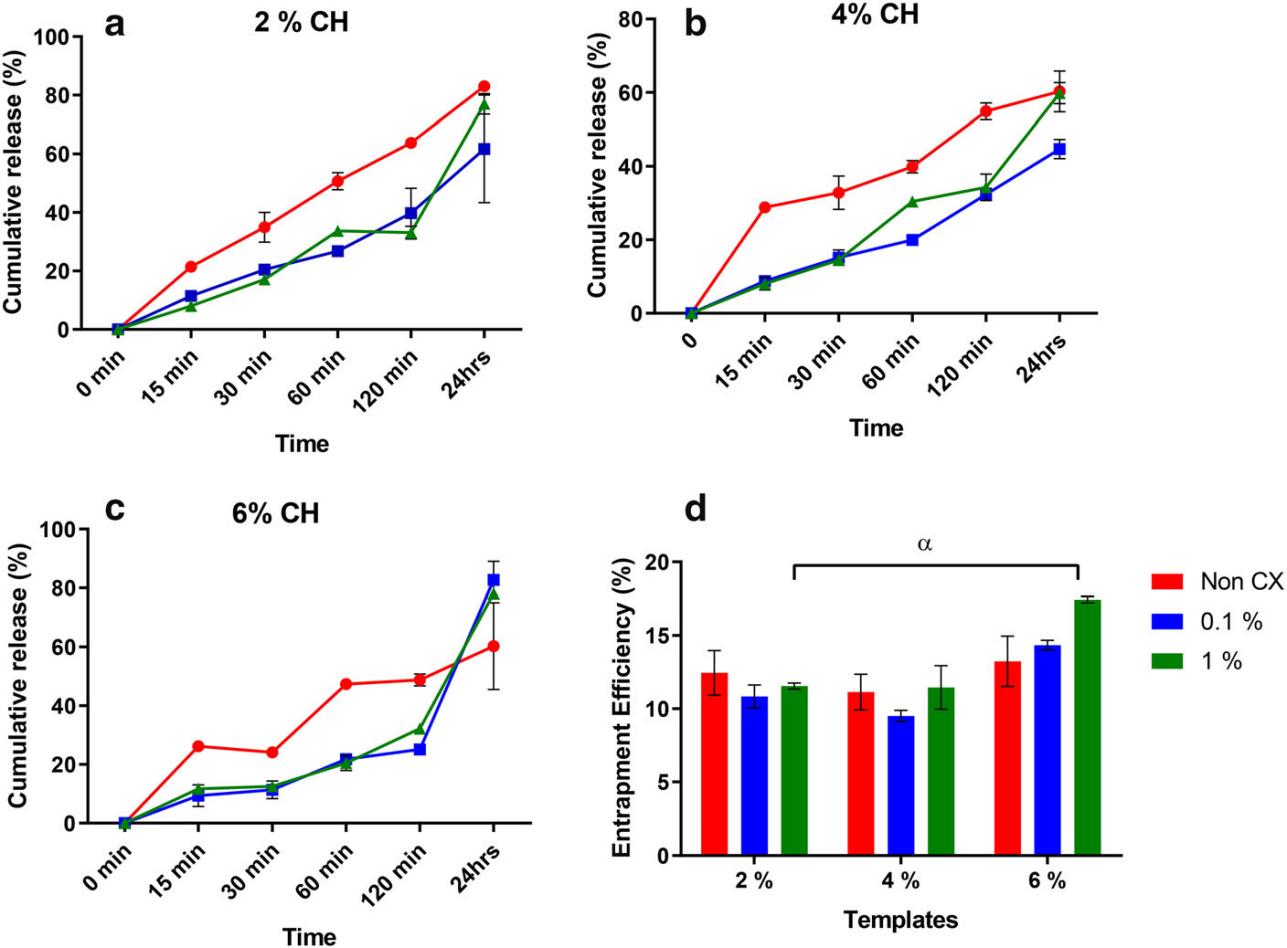
Percentage drug release profile of neat and cross-linked templates at **a** 2%, **b** 4%, **c** 6%, **d** % entrapment efficiency of drug by weight loaded onto the neat and cross-linked templates at 0.1 and 1% glutaraldehyde. Values shown are mean ± SD, where “α”

Drug entrapment efficiency (%) of templates at different concentration of CH and cross-linking agent is represented in Fig. 6. Maximum entrapment is noticed for 6% templates cross-linked at 1% glutaraldehyde (Fig. 6d). This is also coinciding with the higher release profile of the same concentration after 24 h of release (Fig. 6c). A total of 2 and 4% non-cross-linked templates showed a higher percentage of release (Fig. 6 a and b) as compared to the cross-linked counterparts. There is gradual increment in release profile within the initial 2 h of all samples; a higher concentration is noticed at 24 h (Fig. 6 a, b and c).

## DISCUSSION

The concept of this template is to be used either as a core or surface layer in a functionally graded biomimetic membrane for periodontal regeneration with structural and functional gradients as previously reported by Bottino *et al.* (6,30). They had proposed that such scaffolds would exhibit spatiotemporal organisation with multidrug delivery systems in conjunction with bioactive ingredients. Since each layer needs to be graded, structural variations are pivotal investigations that have been conducted on fabricating and characterizing each layer individually (10). More recently, this concept of functionality and layering has also been suggested by Qasim and co-workers (31). Bottino *et al.* (6) used different drugs such as metronidazole and ciprofloxacin in the form of nanofibers (32). Previously, such templates have been synthesized by freeze drying which have certain drawbacks in terms of difficulty in controlling the pore size, low interconnectivity and residual salt and skin formation. Using freeze gelation (freeze casting) can enable to tailor templates with desired porosity by controlling the freezing rate that can be conveniently harnessed (10). Drug release studies using freeze gelated templates have been rarely reported. Therefore, the current investigation delivers insights on such templates to adapt to the functionally graded membrane concept to be either used as a core or surface layer. In this study, we have investigated the effect of cross-linking efficiency on doxycycline hyclate entrapment and release profile. According to the SEM micrographs displayed in Fig. 2, the highly porous and interconnected morphology of the neat templates is clearly visible. Studies conducted on the effect of concentration of CH have reported similar findings (33–35). Another study by Jana and co-workers investigated 4, 6 and 8 wt% solutions. They mentioned that the scaffold porosity decreased as the concentration increased, which was also observed in the current templates (36). A critical parameter in fabricating such templates is the freezing temperature which effects the formation, speed, size and orientation of the solvent crystals (35). Although there have been studies conducted on lyophilizing (freeze drying) CH (37–39) in the past, reports on freeze casting or gelated templates with doxycycline hyclate for use in functionally graded membranes are still lacking. This simple methodology has the tendency to produce highly porous templates as shown by the micro CT results in Table I. Previously, 2% CH solutions were able to produce 85% porosity, which reduced after the addition of hydroxyapatite to 77% (10). In this study, we were able to achieve a 92% porosity with similar concentration, which reduced to 88% and 90% with 4% and 6% concentrations. This could be due to the alterations in the freezing rate (fast or slow cooling) as reported by Yuan and co-workers (40). Interestingly, they also mentioned that cooling rate did not have a significant effect on the porosity of freeze gelled polysaccharide scaffolds. They also observed that larger thermal gradients caused by the high cooling rate resulted in unidirectional morphology (40). Cross-linking of CH-based porous templates with glutaraldehyde has been attempted in the past by different investigators (19,41,42,43). The cross-sectional images showed that the porous morphology was collapsed to some extent. However, Hoffman *et al.* cross-linked CH porous scaffolds at 3.5 wt% and micrographs reported showed that the shape of the pores was nearly round (Table II). The pore size after cross-linking was about 120 to 340 μm with an average of 140 μm. Furthermore, they also mentioned that a reduction in pore size could be observed from 1 to 2.5% CH cross-linked with 0.5% glutaraldehyde and also in the series of 1 up to 2% CH cross-linked with 1% glutaraldehyde (44).

**Table I:** Table Showing the Results of Multiscale Computerized Tomography of 2, 4 and 6% Templates Showing the Porosity Percentage, Pore Size and Structure Thickness

Morphometrical parameters	Value ± Std (μm)		
	2%	4%	6%
Structure thickness	4.62 ± 1.32	4.88 ± 1.46	4.65 ± 2.14
Pore size	78.30 ± 37.65	53.2 ± 24.62	44.91 ± 20.90
Total porosity (%)	92.64	88.48	90.50

**Table II:** Peak Identification of Functional Groups of Chitosan (C_6_H_11_NO_4_)_n_ and doxycycline hyclate (C_24_H_33_CIN_2_O_10_) with their respected references

Wave numbers (cm^−1^)	Peak identification	Reference
Chitosan		
2869	CH_2_ symmetric and asymmetric stretching vibrations	(25)
1657	C=O	(26)
1591	–NH_2_ bending in the amine group	(10)
1419	CH_3_ bending deformation (pyranose ring) (C–H)	(27)
1374	CH_3_ in the amide group, CH bending, CH stretching	(25)
1322		
1151–1026	Glycosidic linkages (symmetric and asymmetric stretching vibration (C–O–C)	(10)
Doxycycline hyclate		
2995–2863	CH_3_ stretching	(28)
1648–1579	C=C stretching	(29)
1455	C–H bending	
1357	CH_3_ bending	
1398	–OH hydroxyl group	
1247–1000	Aromatic in plane and out plane deformation peaks	

A detailed spectroscopic characterisation was conducted to understand the chemical interactions between CH, glutaraldehyde and doxycycline hyclate. CH can react in a number of ways with glutaraldehyde according to an amine catalysed aldol reaction, Michael addition or even as a Schiff’s base pathway. It has mucoadhesive properties mediated by ionic interactions between positively charged amino groups in the biopolymer and negatively charged sialic acid in mucus. Glutaraldehyde can also react with CH and it cross-links in an inter and intra molecular fashion through the formation of covalent bonds specifically with the amino groups of the polysaccharide. However, un-cross-linked CH has high affinity for mucin, whereas cross-linked templates tend to lose this property (45). These are all reliant on the reaction conditions. Figure 3 shows the fingerprint region of cross-linked 2, 4 and 6% CH at 0.1 and 1%. Studies have shown that this interaction is depicted on a spectral profile by disappearance of the NH_2_ band at 1596 cm^−1^ and the formation of the imine band C=N at 1675–1680 cm^−1^ (17,46). The attenuation of the NH_2_ band is assigned to the deprotonation of the ammonium cation and cross-linking with glutaraldehyde. Few other bands at 1400 cm^−1^ are also pointing towards Schiff’s base (46). These results in a more hydrophobic ultrastructure thereby effect the swelling profile although the fingerprint of 2 and 6% after cross-linking were almost similar, 4% showed significant alterations in the glycosidic region. The attenuation of the bands at 1589, 1590 and 1591 is indicative of the deprotonation of the ammonium cations and cross-linking with glutaraldehyde (Fig. 5 b, c and d).

Swelling is the first event during mucoadhesion and in the presence of moisture; swollen CH triggers contact with the mucus layer. This causes mechanical entanglement; therefore, the formation of hydrogen bonds and electrostatic interactions between the polymer and the mucus network is a critical step when formulating GTR membranes (47). Cross-linking of CH templates usually results in a lower swelling percentage. However, in the current study, cross-linking at 0.1 and 1% glutaraldehyde both showed an equilibrium state after 15 min of immersion. This was similar to the profile displayed by non-cross-linked templates. Although the specimens showed alterations in their physical, ultrastructural on visual examination, the swelling profile displayed an unexpected pattern of equilibrium, in between non-cross-linked and cross-linked templates. An investigation conducted by Roberts and Taylor on the interaction of glutaraldehyde and CH reports that a noticeable characteristic of these two material interactions is the yellow brown colour which is indicative of the formation of a chromophore (48). Furthermore, the rates of hydrophilicity and drug release have been reported to be dependent on the surrounding pH. It is speculated by Giri and co-workers that dominant carboxyl groups in the hydrogels would dissociate with an increment of the osmotic pressure inside the hydrogel at higher pH, consequently triggering a rapid release of drug and faster swelling (18).

The *in vitro* release of drug provides an accurate prediction of the release in the environment. A recent review has highlighted that the unique microarchitecture of the periodontal pocket can mimic a “sac” for gels or membranes to reside in them and act as reservoirs for drug release (49); therefore, a conventional dialysis sac was used to perform drug release investigations of the cross-linked templates. Since these FGM will exhibit a unique microarchitectural geometry, chemical, cellular/biochemical composition that needs to be tailored to trigger complex periodontal regeneration (31). The idea was that these scaffolds would become part of a layered membrane with multi-drug templates that can trigger release gradually as they degrade layer by layer thereby being able to deliver a more sustained release profile (10). A 24-h end point could possibly insights on how adjacent layers will need tuning with respect to this layer. Investigations conducted in the past have explored the possibilities of loading tetracycline and doxycycline hyclate on porous CH templates (17,21,41,46,50). It can be speculated that the cross-linked templates showed a sustained release at the completion of 24 h time point. Whilst this sustained release could be considered ideal, it is critical to note that kinetic release is a parameter that is also effected by the percentage of CH (51). Another factor to the sustained behaviour could be due to the glutaraldehyde promoting stronger bonding with doxycycline. The suitable drug release rate was obtained which limits the toxicological effect of the cross-linking agent. The precise mechanism of the slow release of doxycycline from the cross-linked templates as noted in the current investigation is still uncertain and needs further investigation. This can also have an effect on the release profile of the drug. However, mimicking such *in vivo* conditions can be a challenging task whilst studying *in vitro* release. The suitable drug release rate was obtained which limits the toxicological effect of the cross-linking agent. Furthermore, the precise mechanism of the slow release of doxycycline from the cross-linked templates as noted in the current investigation is still uncertain and needs further investigation.

## CONCLUSION

Treating chronic periodontal conditions requires a sustained release of antimicrobial agents and the current study provided a clear insight into the ability of freeze casted chitosan templates to be adapted in a functionally graded template for guided tissue regeneration. The release of drug from the non-cross-linked specimens was higher as compared to cross-linked templates. The higher concentration of chitosan and glutaraldehyde permits a higher drug-loading tendency. Data obtained is indicative of the possibility of using cross-linked freeze casted chitosan templates as drug carriers for sustained drug release.
